# Probiotics, Anticipation Stress, and the Acute Immune Response to Night Shift

**DOI:** 10.3389/fimmu.2020.599547

**Published:** 2021-01-28

**Authors:** Nicholas P. West, Lily Hughes, Rebecca Ramsey, Ping Zhang, Christopher J. Martoni, Gregory J. Leyer, Allan W. Cripps, Amanda J. Cox

**Affiliations:** ^1^ School of Medical Science and Menzies Health Institute QLD, Griffith University, Gold Coast, QLD, Australia; ^2^ Menzies Health Institute QLD, Griffith University, Gold Coast, QLD, Australia; ^3^ UAS Laboratories, Windsor, WI, United States; ^4^ School of Medicine and Menzies Health Institute QLD, Griffith University, Gold Coast, QLD, Australia

**Keywords:** night shift, DDS-1, UABla-12, immunity, anticipatory stress

## Abstract

**Introduction:**

Sleep disturbance and sleep disruption are associated with chronic, low grade inflammation and may underpin a range of chronic diseases in night shift workers. Through modulation of the intestinal microbiota, probiotic supplements may moderate the effects of sleep disruption on the immune system. The aim of this study was to examine 14 days of daily probiotic supplementation on the acute response of acute phase proteins and immune markers to sleep disruption associated with night shift work (Australia and New Zealand Clinical Trials Registry: 12617001552370).

**Methods:**

Individuals (mean age 41 ± 11 yrs; 74% female) performing routine night shift were randomly assigned to a probiotic group (1 × 10^10^ colony forming units (CFU) *Lactobacillus acidophilus* DDS-1 or 1 × 10^10^ CFU *Bifidobacterium animalis* subsp. *lactis* UABla-12) or placebo (n= 29 per group). Participants undertook a 14-day supplementation period that coincided with a period of no night shifts followed by two consecutive night shifts. Blood samples were collected prior to the start of supplementation (V1), prior to commencing the first night shift (V2), after the first night shift (V3) and after the second night shift (V4). Serum was assessed for markers of stress (cortisol), acute phase response (C reactive protein (CRP), erythrocyte sedimentation rate, pentraxin), adhesion markers (serum E-selectin, mucosal vascular addressin cell adhesion molecule 1 (MAdCAM-1), and serum cytokines (interleukin (IL)-1ra, IL-1β, IL-6, tumor necrosis factor (TNF)-α, IL-10). Sleep quality was assessed with the Pittsburgh Sleep Quality Index (PSQI) and a Fitbit activity tracker.

**Results:**

The groups were well balanced on key markers and the probiotic strains were well tolerated. The 14-day supplementation period that coincided with typical night-day sleep-wake cycles leading up to night shift (V1 to V2) was associated with significant changes in the placebo group in the concentration of serum cortisol (p = 0.01), pentraxin (p = 0.001), MAdCAM-1 (p = 0.001), and IL-1ra (p=0.03). In contrast, probiotic supplementation moderated changes in these serum markers from V1 to V2. No significant interaction effects (time by group) were observed for the serum markers prior to and after night shift work following probiotic supplementation due to the substantial changes in the serum markers that occurred during the normal sleep period from V1 to V2.

**Conclusions:**

Probiotics may moderate the effects of anticipatory stress on the immune system in the lead up to night shift.

## Introduction

Night shift work is associated with a range of adverse health outcomes, including obesity and metabolic disease, cardiovascular disease and depression (1). Night shift related impacts on health and wellbeing are being recognized within the framework of a sleep loss epidemic, with even minor sleep loss and fragmented sleep having negative consequences on physiological health, mental health and performance (2). Given the growing evidence of the negative impacts of fragmented and disrupted sleep and night shift on health, there is increasing focus on strategies to address the negative impacts of sleep disruption.

In recent years the role of chronic low-grade inflammation has been recognized as a biological driver of ill health associated with stress (3). Sleep deprivation and sleep loss have been reported to be associated with changes in multiple immune parameters that favor a pro-inflammatory state, which may underlie the negative health effects of sleep disruption. For example, total sleep deprivation for 40 h in 19 healthy men and women was associated with increases in adhesion markers and pro-inflammatory serum cytokines (4). Additionally, restricting sleep to <6 h per night for a week in eight healthy volunteers (50% female) has been reported to decrease neutrophil phagocytic activity and alter the ratio of the interferon(IFN)-γ induced T-cell and NK-cell attractant chemokines CXCL9/CXCL10 and CCL5/CCL9 and decrease the frequency of circulating CD4^+^ T-cells (5). Lastly, a 4-day simulated night shift protocol in which blood samples were collected over two 24-h periods from eight healthy individuals reported a significant reduction in diurnal transcripts related to NK cell responses and the Jun/AP1 and STAT pathways (6). This evidence of sleep-induced immune perturbations along with evidence that pathogen-induced immune responses can negatively impact sleep architecture (7), highlight a bidirectional relationship between sleep and immunity which may have profound implications for diseases underpinned by immune dysregulation.

Sleep deprivation and sleep loss alters the physiological systems that drive diurnal patterns of immune cell distribution and cytokine activity. A normal day-night sleep-wake cycle has a dynamic role in regulation of the hypothalamic-pituitary-adrenal axis (HPA axis) that controls circadian rhythm. Systemic levels of key immune regulatory cytokines exhibit a circadian profile with peaks at mid-morning and late afternoon (8), reflecting the secretion of neuropeptides, in particular glucocorticoids (9). Interestingly, disruption to the pattern of activity of the HPA axis alters the mobilization of stem cells that may also impact the immune system (10). Changes in normal sleep-wake patterns alters inflammatory processes through effector systems of the central nervous system.

The microbiota plays a key role in the ontogeny of the immune system and the regulation of inflammation throughout life. Recognition that direct interaction of commensal bacteria with epithelial cells modulates the mucosal cytokine milieu and that microbial metabolites induce regulatory T-cells (11–13) has led to interest in modifying the microbiome to influence immune homeostasis. Probiotics are able to transiently colonize the gut microbiota (14, 15) and are postulated to exert beneficial effects on health by modifying the immune system. Research from our group and others provides evidence that some probiotics are able to reduce the risk of common respiratory illnesses (16–18) and modulate the immune system (19, 20). Evidence of the health effects and immune modulating capacity of probiotics suggests there is promise in investigating the use of a probiotic supplement to ameliorate sleep disruption-induced changes in inflammation associated with night shift.

This aim of this study was to investigate the acute effects of two independent probiotic strains, *Lactobacillus acidophilus* DDS-1 and *Bifidobacterium animalis* subsp. *lactis* UABla-12, on indices of the immune system in individuals undertaking night shift work. *L. acidophilus* DDS‐1 and *B. lactis* UABla-12, alone or in combination, have previously been shown to reduce abdominal pain severity and symptomology in irritable bowel syndrome (21), normalize bowel habits in functional constipation (22), provide abdominal symptom relief in lactose intolerance (23) and demonstrate an immune regulatory role in atopic dermatitis (24) and respiratory tract infection (25) in randomized controlled trials.

In a surprise finding in the current study, the anticipation of undertaking night shift was associated with a number of statistically significant perturbations in key stress and immune markers, with the magnitude of the changes moderated by probiotic supplementation. These findings have important implications for understanding the role of anticipatory stress on inflammation and health and suggest there may be broader health implications associated with night shift than just circadian disruption. Importantly, our findings provide initial evidence for the use of these probiotic strains to moderate the effects of stress associated with undertaking night shift.

## Materials and Methods

### Study Design

**Figure 1:**
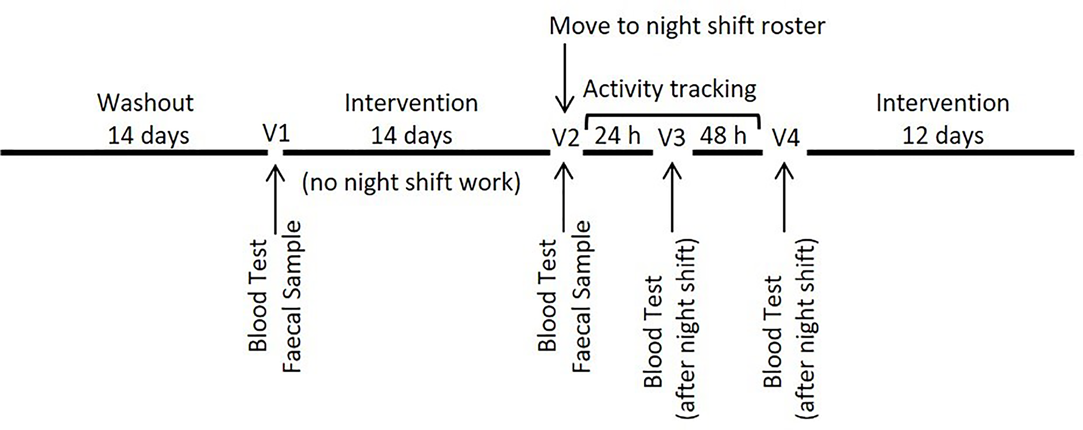
Study design.

This was a randomized double-blind three-arm parallel-group trial in which participants were allocated to one of three groups; one of two probiotic supplement groups or a placebo group. Following recruitment, participants were required to complete an initial 14-day washout period during which they were required to refrain from the consumption of any probiotic supplements, adhere to a typical night-day sleep-wake cycle and complete a series of health and demographic questionnaires, including the Connor Davidson Resilience Scale. Following the washout period, participants were randomized and commenced supplementation for a period of 14 days prior to commencing an altered sleep-wake cycle with two nights of nightshift work. Participants continued supplementation throughout two nights of sleep disruption and for an additional 12 days (during which no specific work schedule was required). Blood samples were collected at baseline (V1) (before probiotic intervention), before (V2) and after (V3) the first night of sleep disruption and again after the second night (V4) of sleep disruption (**Figure 1**). A fecal sample and three-day diet diary were collected at V1 and again at V2 to determine recovery of the probiotic bacteria and compliance leading into the night shift period. Sleep quality was assessed using the Pittsburgh Sleep Quality Index (PSQI) at the beginning (V1) and end of the intervention period (V4). Participants also wore a Fitbit (Fitbit, San Francisco, USA) activity tracker to allow monitoring of activity and sleep data. The study was approved by the Griffith University Human Research Ethics Committee (2017/646) and the Gold Coast Hospital and Health Service Ethics Committee (HREC/17/QGC/271).

### Participants

Individuals undertaking night shift work, including medical staff, transport, emergency services and security workers were contacted by email alerts with the invitation to participate in the research. Participants were required to be between 18 and 65 years of age and engaged in a rotating work roster that included night shift and day shift work. Individuals using immune-modulatory medications, sleep medications, antibiotics, who had taken probiotics in the month prior to the study or who had gastrointestinal or metabolic conditions, were pregnant or had a current bacterial or viral illness were excluded from participation.

### Supplementation

Participants were required to consume one capsule daily (not less than 1 × 10^10^ colony forming units; cfu) of either *Lactobacillus acidophilus* (DDS-1), *Bifidobacterium animalis* subsp. *lactis* (UABla-12) or a placebo, identical in appearance. Compliance was assessed with a daily checklist and the return of the remaining capsules for counting at the end of each participant’s study period.

### Blood Sample Collection and Analysis

Blood samples were collected between 06:00 and 09:00 hours on the sample collection days. Blood samples were collected into K3EDTA tubes (Greiner Bio-one; Frickenhausen, Germany), serum separator (BD Vacutainer^®^ (BD, Franklin Lakes, NJ, USA) and heparinized tubes (Greiner Bio-one; Frickenhausen, Germany) and PaxGene tubes (Qiagen, Hilden, Germany). Plasma and serum were separated by centrifugation at 3500*g* for 10 min and stored frozen at −80°C until analysis.

Determination of C-reactive protein (CRP) concentrations was completed using a COBAS Integra 400 system and commercially available reagents, calibrators and controls (Roche Diagnostics). Concentrations of other soluble analytes, including lipopolysaccharide binding protein (LBP; Raybio^®^, GA, USA), intestinal fatty acid binding protein (iFABP; Hycult^®^ Biotech), pentraxin (RnD Systems, Minneapolis, MN, USA), serum alpha amyloid (SAA; RnD Systems, Minneapolis, MN, USA), E-selectin (eBioscience, Vienna, Austria, mucosal vascular addressin cell adhesion molecule 1 (MAdCAM-1; RnD Systems, Minneapolis, MN, USA); interleukin(IL)1β, IL-6, IL-10, IL-1ra, tumor necrosis factor-alpha (TNF-α), and cortisol were determined using commercially available enzyme-linked immunosorbent assays (ELISA) and suspension array kits (Merck Milliplex, Darmstadt, Germany) according to the manufacturer’s instructions. Dilution factors were optimized for each assay to ensure an acceptable goodness of fit to the standard curve. For each analyte, samples were assessed in duplicate (typical intra-assay coefficients of variation were <6.5%) and all samples from a single individual were assessed on a single assay plate. Inter-assay coefficients of variation ranged from 6.6% to 13.3%.

### Fecal Sample Collection and Analysis

A fecal sample and 3-day food diary were collected from each participant prior to supplementation and at day 14. Participants were provided with a collection kit with instructions not to contaminate the sample with urine or water and to store the sample at room temperature until their study visit. Stool samples were frozen at −80°C upon receipt until processing. For probiotic bacteria identification, samples were brought to room temperature and total DNA was extracted from 2.0 g of fecal material using the QIAGEN DNA Stool Mini Kit (Qiagen, Hilden, Germany) according to the manufacturer’s instructions. Following extraction, DNA concentrations were determined using a Nanodrop Spectrophotometer (Thermo Scientific, Scoresby, VIC, Australia). Isolated fecal DNA was used for the detection of the bacterial species *B. lactis* and *L. acidophilus* in the supplement. Separate real-time polymerase chain reactions (PCR) were used for microbial identification. Sequence specific primers and probes were used as described previously (22). Reactions included Universal Fast PCR Master Mix (Life Technologies, Carlsbad, CA, USA), 10 µM of each of forward and reverse primer and probe in a 10-µl reaction volume. Cycling conditions were 50°C for 2 min, 95°C for 20 s, and 40 cycles of 95°C for 5 s, 48°C for 15 s and 60°C for 20 s for *L. acidophilus* and 50°C for 2 min, 95°C for 20 s, and 40 cycles of 95°C for 5 s, 52°C for 15 s and 60°C for 20 s *B. lactis*. An additional reaction using a pair of universal bacteria primers and probe (26) was also performed for each sample again using Universal Fast PCR Master Mix (Life Technologies, Carlsbad, CA, USA), 10 µM of each of forward and reverse primer and probe in a 10-µL reaction volume with cycling conditions 50°C for 2 min, 95°C for 20 s, and 40 cycles of 95°C for 5 s, 55°C for 15 s, and 60°C for 20 s. The mean cycle threshold (C*_T_*) value was determined for duplicate samples and the change in C*_T_* values (ΔC*_T_*) between pre- and post-intervention visits determined. For the *L. acidophilus* and *B. lactis* reactions, the ΔC*_T_* was normalized against the ΔC*_T_*value obtained**from the universal bacterial reaction to standardized for sample input and converted to a fold change value.

### Statistical Analysis

Statistical analyses were performed with IBM SPSS Statistics for Windows (IBM Corporation, Armonk, NY) and GraphPad Prism Version 7.00 for Windows, (GraphPad Software, CA, USA). Given that substantial inter-individual variability is reported in the literature for serum cytokines and other soluble immune markers (27) our sample size was based on relevant published research on sleep and soluble immune markers (28, 29) and the effects of probiotic supplements on immune markers (30). Data are reported as mean ± standard deviation (SD) or mean % change and 95% confidence interval unless otherwise indicated. Distributions of all markers (questionnaire and immune) were assessed for normality and transformed where appropriate. Differences in demographic and immune data (**Table 1**) at baseline were assessed with a one-way ANOVA for continuous variables, or a Pearson Chi Square test for categorical variables. The effect of supplementation was analyzed using a two-factor ANOVA (time × group). Any significant interaction effects (*F* ratios) were assessed using a Student’s paired t-test. Post-hoc analysis was undertaken using a Student’s t-test or appropriate non-parametric tests. Statistical significance was accepted at p=0.05.

**Table 1:** Baseline demographic measures between the groups.

	DDS-1	UABla-12	PLA	p-value*
per protocol completions (n)	29	29	29	
F/M (n)	22/7	21/8	21/8	0.94^c^
Age (years)	41.8 ± 11.2	40.7 ± 12.6	41.6 ± 11.3	0.94
BMI (kg/m^2^)	25.1 ± 4.8	24.3 ± 7.3	29.4 ± 6.1	0.74
Occupation				
Healthcare	20/29	21/29	23/29	0.59^c^
Emergency Services	4/29	6/29	5/29	
Transportation	2/29	1/29	1/29	
Other	3/29	1/29	0/29	
WCC (×10^9^/L)	6.0 ± 1.5 (5.6, 3.5–8.9)	5.6 ± 1.6 (5.6, 3.2–9.0)	6.3 ± 1.9 (6.1, 3.3–10.8)	0.31
CRP (mg/L)	1.8 ± 2.3 (0.7, 0.1–8.7)	1.8 ± 2.1 (0.7, 0.1–7.6)	1.6 ± 1.9 (1.0, 0.0–7.5)	0.97^†^
CRP (n)				
<1 mg/L	15	16	16	0.94^c^
1–3 mg/L	9	7	9	
>3 mg/L	5	6	4	
LBP (µg/ml)	4.6 ± 1.7 (4.1, 2.6–10.1)	4.3 ± 1.5 (4.3, 1.8–7.9)	4.0 ± 1.4 (4.2, 1.7–6.9)	0.30^†^
iFABP (ng/ml)	1.52 ± 1.07 (1.3, 0.4–6.0)	1.30 ± 0.61 (1.3, 0.4–3.4)	1.88 ± 1.58 (1.4, 0.7–8.5)	0.17^†^
Pentraxin (µg/ml)	1.78 ± 0.96 (1.39, 0.73–5.46)	1.22 ± 0.47 (1.27, 0.61–2.15)	1.36 ± 1.13 (1.05, 0.62–6.73)	0.008^†^
SAA-1 (vg/ml)	3.04 ± 2.71 (2.03, 0.50–12.92)	2.71 ± 2.96 (1.91, 0.45–14.05)	3.21 ± 2.51 (2.35, 0.45–9.84)	0.50^†^
MAdCAM-1 (ng/ml)	5.80 ± 1.61 (5.70, 3.05–9.32)	5.97 ± 2.61 (5.60, 3.38–17.67)	5.37 ± 1.38 (5.18, 2.87–8.81)	0.52^†^
sE-selectin (ng/ml)	35.7 ± 16.1 (36.4, 11.7–77.6)	31.4 ± 13.3 (28.3, 11.8–68.5)	38.1 ± 19.4 (30.8, 13.5–79.8)	0.46^†^
Cortisol (µg/dl)	13.6 ± 6.4 (12.0, 7.1–52.4)	12.5 ± 6.1 (10.6, 5.8–36.6)	11.4 ± 3.7 (10.3, 6.4–239.3)	0.69^†^

Data are presented as mean ± SD (median, range) using raw data.

Ng, nanograms; ml, milliliter; µg, microgram; kg, kilogram; L, liter; mmol, millimole; m, meters; WCC, white cell count; CRP, C-reactive protein; LBP, lipopolysaccharide binding protein; iFABP, intestinal fatty acid binding protein; SAA, serum alpha amyloid; MAdCAM-1, mucosal vascular addressin cell adhesion molecule 1.

*for one-way ANOVA; ^†^using transformed data; ^c^p-value based on Chi-squared analysis of frequencies.

## Results

### Baseline Data

A total of 94 participants were recruited to the study and 87 participants (n=29 in each group) were included in the final analysis, as detailed in the CONSORT diagram (**Supplementary Figure 1**). The groups were well matched on gender, age, body mass index (BMI), and occupation (**Table 1**). Overall, the supplements were well tolerated with high levels of compliance based on self-reported number of doses missed (DDS-1: 0.4 ± 1.1 d; UABla-12: 1.3 ± 2.5; PLA: 0.5 ± 1.1). A significant difference between the groups at V1 was observed in sleep quality metrics as assessed by the PSQI (**Table 2**). Baseline sleep scores as measured by the PSQI were a significant ~30% lower (p=0.02) in the DDS-1 group compared to the UABla-12 group (**Table 2**). Categorization of participants into good and poor sleepers based on their PSQI was comparable between the groups and no differences between the groups was observed in average sleep time or average steps per day *via* Fitbit monitoring per day at V1 (**Table 2**). A significant difference between the groups was observed for the concentration of serum pentraxin, with the concentration ~32% higher (p=0.005) in the DDS-1 group compared to the UABla-12 group, but groups were otherwise matched on baseline assessment of soluble markers.

**Table 2:** Differences between the groups at V1 in sleep quality based on the PSQI and in sleep minutes and steps as measured by fitbit.

	DDS-1	UABla-12	PLA	p-value
PSQI^‡^	5.6 ± 2.2	7.9 ± 3.5	6.9 ± 2.6	0.04^*^
Sleep categorization (good/poor)	12/17	10/19	9/20	0.70^c^
Fitbit sleep time (min)	396 ± 66	405 ± 65	411 ± 73	0.78^*^
Fitbit steps/day^a^	9809 ± 3308	9628 ± 3417	9740 ± 3719	0.98^*^

Data are presented as mean ± SD. ^‡^out of a maximum score of 21; *for one-way ANOVA using transformed data; ^c^p-value based on Chi-squared analysis of frequencies.

^a^For each individual a daily average was determined based on all available data from the Baseline period.

### Fecal Recovery

Fecal samples were collected at V1 and V2 with changes in the recovery of the probiotic species assessed as a surrogate measure of compliance with supplementation. A difference in the recovery of *L. acidophilus* DDS-1 in the DDS-1 group (median increase: 7.7-fold post-supplementation) was noted compared to the UABla-12 (median increase: 1.5-fold) and PLA (median increase: 1.8-fold) groups. Similarly, a difference in the recovery of *Bifidobacterium lactis* (UABla-12) in the UABla-12 group (median increase: 1700-fold post-supplementation) was noted compared to the DDS-1 group (median change: 80% decrease) and the PLA group (median increase: 1.3-fold). Large inter-individual variation in the recovery of both DDS-1 and UABla-12 was noted.

### Effects on Serum Hormones, Acute Phase Proteins, and Immune Markers

**Figure 2:**
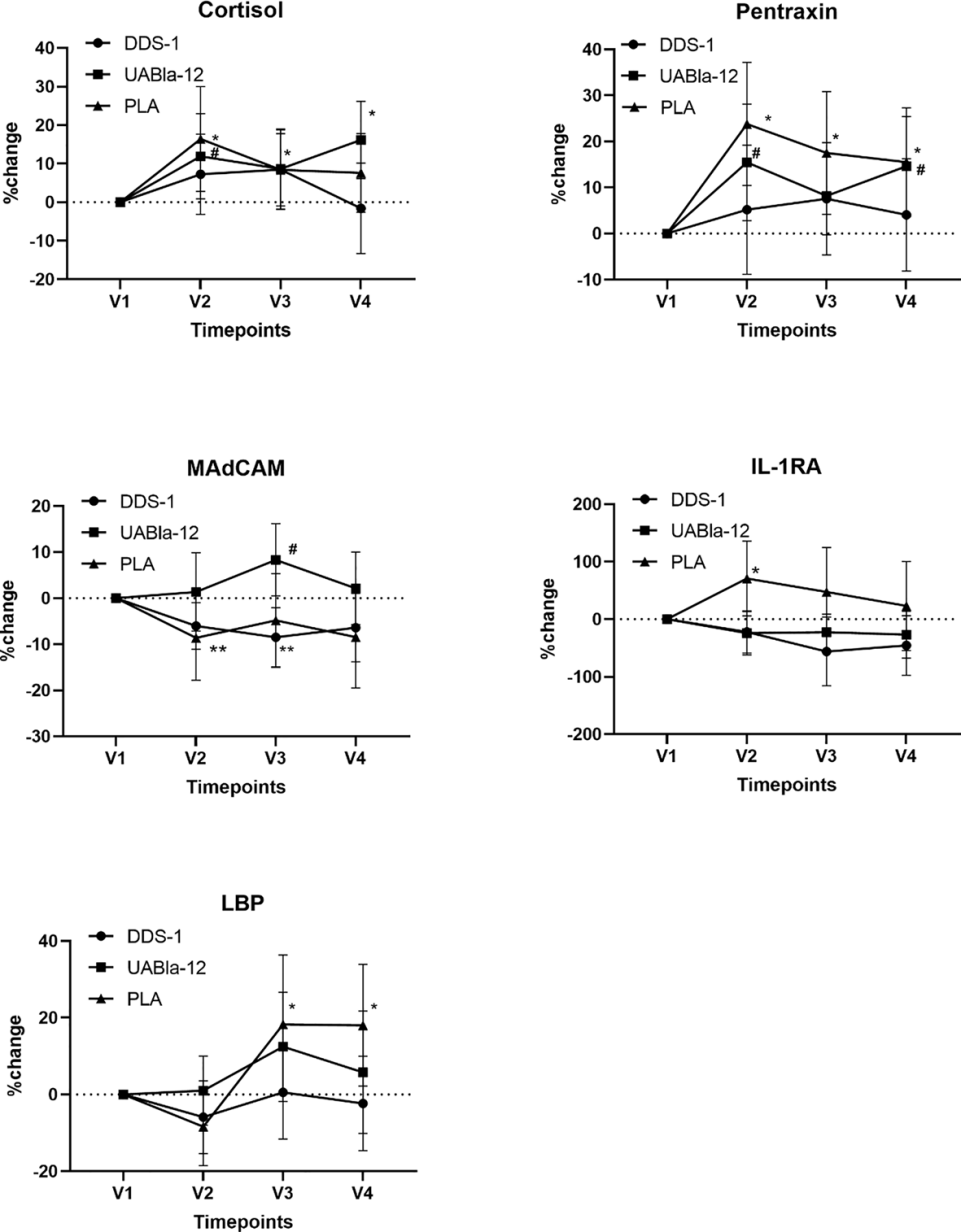
Changes in the concentration of serum analytes over the course of the study. Significantly larger within group changes in the placebo group are evident in all analytes from V1 to V2. Data are % change and 95% CI. The large changes in these analytes while adhering to typical night-day sleep-wake cycle were greater than the effect of nightshift on indices of stress, the acute phase response, serum cytokines and intestinal integrity markers. *significant change from V1 in the placebo group, ^#^significant change from V1 in UABla-12 group. **significance chance from V1 in the DDS-1 group.

Probiotic supplementation appears to have moderated changes in a number of serum analytes between V1 and V2. As shown in **Figure 2**, compared to the probiotic groups, the placebo group had a significantly larger within group time effect from V1 to V2 in the concentration of cortisol (PLA, 16%; p = 0.01; DDS-1, 7%; p = 0.16; UABla-12, 11%; p = 0.03), pentraxin (PLA, 23%; p = 0.001; DDS-1, 7%; p = 0.45; UABla-12, 15.5%; p = 0.02), MAdCAM-1 (PLA, −8.6%; p = 0.05; DDS-1, −6%; p = 0.02; UABla-12, 2%; p = 0.75), IL-1ra (PLA, 71%; p = 0.03; DDS-1, −22%; p = 0.22; UABla-12, −24%; p = 0.19) and LBP (PLA, −8%; p = 0.09; DDS-1, −6%; p = 0.22; UABla-12, 2%; p = 0.86). No between group differences were found for the markers over the period of nightshift.

### Effects on Sleep Quality

**Figure 3:**
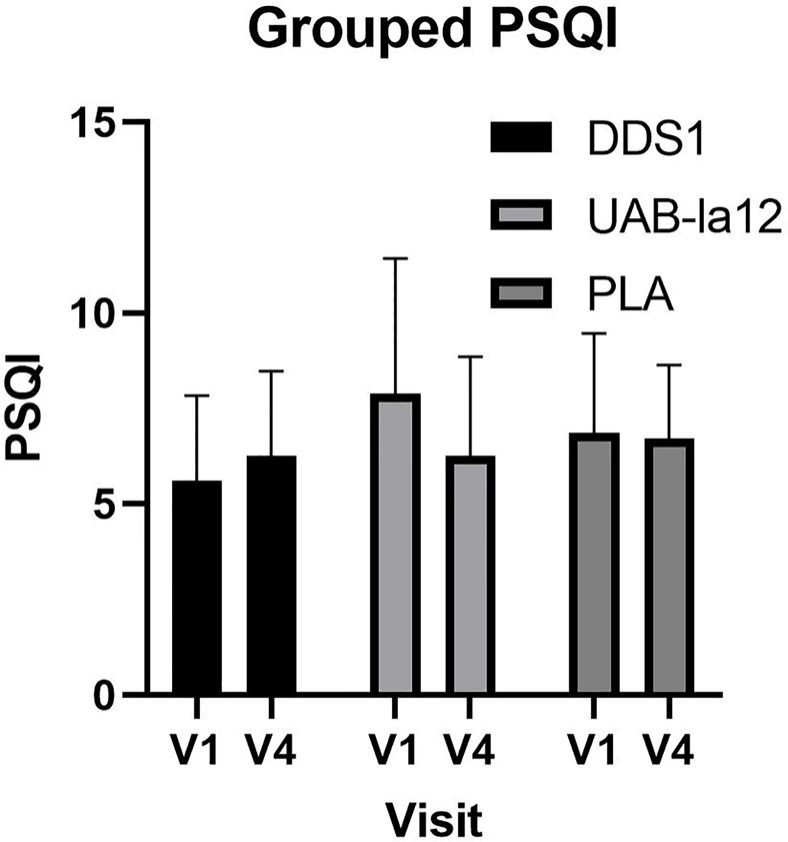
Changes in the PSQI from pre-supplementation (V1) to the end of supplementation (V4). A statistical trend for a 22% decrease in the UABla-12 group was observed (p=0.063). Data is median and 95% confidence intervals.

A significant time by group effect was observed for changes in the PSQI between pre-supplementation (V1) and end (V4) of supplementation (F(2,62) = 4.32; p = 0.018). Post-hoc analysis revealed that UAB-la12 was associated with a statistical trend for a 22% (p = 0.06) decrease in the PSQI (**Figure 3**). There was a statistical trend for an inverse correlation at baseline of the PSQI and the Connor Davidson Resilience Scale (r = −0.21, p = 0.07).

## Discussion

The aim of this study was to investigate the acute effects of probiotic supplementation on indices of stress, the acute phase response and inflammation during two nights of night shift. While no between group differences over the course of the study were observed in the serum analytes, there were a number of significant within-group changes observed between V1 and V2, with the largest changes observed in the placebo group. The period between V1 and V2 was when patients were supplementing in the lead up to night shift and adhering to a night-day sleep-wake cycle. The effects observed between V1 and V2 are consistent with an anticipatory stress effect prior to the start of night shift (31). Anticipatory stress refers to the stress associated with upcoming events (32) and is linked to disease (33) and dysregulation of the HPA axis and the immune system (34). The within-group differences provide early evidence that DDS-1 and UABla-12 may ameliorate the biological impacts of this stress, and, that UABla-12 may also improve sleep quality. High rates of recovery of the ingested probiotic species were observed in the supplement groups, although large variation between subjects was observed. Further, while both probiotic supplements contained identical numbers of colony forming units, the UABla-12 strain elicited a greater fold-change in fecal recovery. This research provides a new paradigm for understanding the role of probiotic supplementation in stress-induced perturbations of the immune system.

Given our study involved healthy individuals, the 10% to 70% changes in the concentration of a range of serum markers, in particular cortisol, pentraxin, MAdCAM-1, IL-1ra, and LBP, across all three groups during a typical night-day sleep-wake cycle (between V1 and V2) was unexpected. Further to this, the anticipation-induced changes in the serum markers was greater than that induced by night shift; an outcome that limits interpretation of other differences between the groups following night shift. The changes in serum markers in the lead up to night shift is consistent with understanding of anticipatory stress on sleep, hormones and immune indices (31, 32). Anticipatory night shift stress is also associated with changes in the autonomic nervous system and anxiety (31). Interestingly, probiotic supplementation appears to have moderated the magnitude of the change across these key markers of stress, the acute phase response and the immune system. Our observation that night shift did not induce changes in serum cytokines is in contrast with other research. Sleep deprivation in humans has been shown to alter serum cytokine concentration, the expression of toll like receptors and adhesion markers (4, 35). While research has shown that probiotics and modifying the gut microbiota can reduce the effects of acute and chronic stress the authors are unaware of other research examining a role for probiotics in anticipatory stress.

This study confirms previous findings that DDS-1 and UABla-12 are able to be delivered to the gut and be recovered in feces (22). Delivery of probiotic supplements to the gut is considered essential for probiotics to exert influence on the mucosal immune system. Animal studies provide evidence for interaction between probiotics entering the large bowel and the microbiota and gut immune system. In pigs, probiotic supplementation increased the diversity of the microbiota and altered gene expression of key innate immune molecules and cytokines (36). A range of immunomodulatory effects of probiotics in the gut have also been described in mice (37, 38). Interaction between probiotic strains and the gut immune system is recognized to be an important step in the mechanism linking probiotic supplementation to beneficial health effects. An interesting finding is the difference between the probiotic supplement groups in the recovery of the respective species, with the UABla-12 species recovered in orders of magnitude higher amounts than the DDS-1. Factors that influence the recovery of probiotic bacteria include the supporting matrix and encapsulation, and resistance to stomach acids. As yet, there is little information on dose response and fecal recovery of probiotic strains (39). Other factors related to dietary intake and intestinal function, such as gut motility, may also impact survival and recovery of ingested species, and may contribute in part to the large inter-individual responses observed in the study. The oral microbiome also plays a role in gut immune regulation and may impact on the immunoregulatory effects of probiotic supplements. In mouse models the oral microbiome is able to modify the balance of Th1/Th2, Th17 and macrophage immune cells in the gut (40). The impact of various supplement-specific factors and host factors on probiotic bacteria survival, highlight the complexity of a generic supplementation strategy, particularly given that lower doses of some supplements are able to elicit immunomodulation (41). Improved understanding of intestinal physiology may yield information on the factors underlying individual responses in the patterns of recovery of probiotic strains to guide supplementation strategies that can be confirmed through dose response studies. The results here are nevertheless consistent with a higher fecal recovery of the *Bifidobacterium* genus (over *Lactobacillus*), in part due to a more favorable environment of the large intestine (42, 43).

A pilot discovery study and meta-analyses have shown that probiotics may be able to improve sleep quality (44, 45). Furthermore, microbial profiling of the gut using 16s rRNA sequencing in bipolar disorder indicates that *Bifidobacterium* or *Lactobacillus* counts may play a role in sleep (42). At baseline the average PSQI score for all three groups had ranked them in the poor sleep category, with approximately two thirds of each group recording poor sleep scores (PSQ>5). This indicates that the effects of anticipatory stress in the lead up to nightshift and the moderation of these effects by the probiotic supplements are on top of the deleterious effects of poor sleep. Poor sleep is also associated with higher symptoms of depression and lower resilience. At baseline we found a moderate correlation between the PSQI and resilience as measured by the 10 item CD-RISC. Associations between the CD-RISC and sleep quality are reported in healthy (46) individuals, groups with disease (47) and in individuals undertaking shift work (48), with similar correlations as observed in the present study. Animal and human work demonstrate that probiotics have beneficial effects for mental health and sleep quality (44, 49). In our study, the UABla-12 group exhibited a trend for improved sleep quality from V1 to V4 consistent with effects reported in a meta-analysis examining probiotic supplementation and sleep (45).

The gut microbiota plays a role in Toll‐like receptor signaling pathways, and in turn the immune system (50). *L. acidophilus* DDS‐1 was previously shown to help modulate the fecal and mucosal microbiota in pre-clinical models, while downregulating inflammatory cytokines in serum and colonic explants (15, 51). Additionally, a probiotic blend of *B. lactis* UABla‐12 and L. acidophilus DDS‐1, resulted in improved atopic dermatitis scoring in young children, with modulation of blood lymphocyte subsets (i.e., CD4, CD8 and CD25) suggested as a potential mechanism (24). In addition to the above, the microbiota can effect visceral afferent pathways (52), and lactic acid bacteria have been shown to modulate μ‐opioid receptor expression and activity (53–55), as well as intestinal serotonin levels and transporter expression (56).

A number of limitations to the study should be acknowledged. While conducted in a community setting improves ecological validity, the variability in baseline PSQI scores and in the soluble immune markers may have limited our understanding of nightshift-induced changes in immune analytes given the relationship between sleep and inflammation. Recent commentaries note the need for the probiotic field to focus on individuals with less heterogeneity in key biological or disease markers to better discern the effect of supplementation (57). The current study did not include global microbial compositional profiling and so it is unclear if differences in microbial diversity or in specific species being present at baseline accounts for the variability in the recovery of the supplemental strains and other effects. Questions also remain on the required duration of supplementation to elicit change in immune markers. In conclusion, supplementation with 10 billion CFU daily of DDS-1 and UABla-12 was well tolerated and resulted in strong fecal recovery of both strains, an important initial requirement for the beneficial effects of probiotics. Probiotic supplementation moderated the impact of anticipation stress on the immune system. While the large changes in circulating hormones and immune markers in response to the anticipation of night shift precluded an assessment of the effects of probiotics on sleep-induced inflammation, the changes observed in the experimental groups in the lead up to night shift indicate that probiotic supplementation may be a strategy to address the acute effects of anticipatory stress.

## Data Availability Statement

The raw data supporting the conclusions of this article will be made available by the authors, without undue reservation.

## Ethics Statement

The studies involving human participants were reviewed and approved by Griffith University Human Research Ethics Committee (2017/646) and the Gold Coast Hospital and Health Service Ethics Committee (HREC/17/QGC/271). The patients/participants provided their written informed consent to participate in this study.

## Author Contributions

Study design and concepts: NW, AJC, AWC, GL, CM. Data analysis and interpretation: NW, AJC, AWC, PZ, RR, LH. All authors contributed to the article and approved the submitted version.

## Funding

This study was funded through a grant to Griffith University by UAS Laboratories.

## Conflict of Interest

Authors GL and CM are employed by UAS Laboratories LLC.

The authors declare that this study received funding from UAS Laboratories LLC. The funder had the following involvement with the study: Input into the study design and manuscript drafting. The funder was not involved in the collection of samples and data, sample and data analysis or the interpretation of data.

## Supplementary Material

The Supplementary Material for this article can be found online at: https://www.frontiersin.org/articles/10.3389/fimmu.2020.599547/full#supplementary-material
