# Corrigendum: Probiotics, Anticipation Stress, and the Acute Immune Response to Night Shift

**DOI:** 10.3389/fimmu.2021.713237

**Published:** 2021-06-21

**Authors:** Nicholas P. West, Lily Hughes, Rebecca Ramsey, Ping Zhang, Christopher J. Martoni, Gregory J. Leyer, Allan W. Cripps, Amanda J. Cox

**Affiliations:** ^1^ School of Medical Science and Menzies Health Institute QLD, Griffith University, Gold Coast, QLD, Australia; ^2^ Menzies Health Institute QLD, Griffith University, Gold Coast, QLD, Australia; ^3^ UAS Laboratories, Windsor, WI, United States; ^4^ School of Medicine and Menzies Health Institute QLD, Griffith University, Gold Coast, QLD, Australia

**Keywords:** night shift, DDS-1, UABla-12, immunity, anticipatory stress

In the original article, there was a mistake in **Figure 2** as published. The asterisks to denote significance do not appear in the correct place. The corrected **Figure 2** appears below.

**Figure 2:**
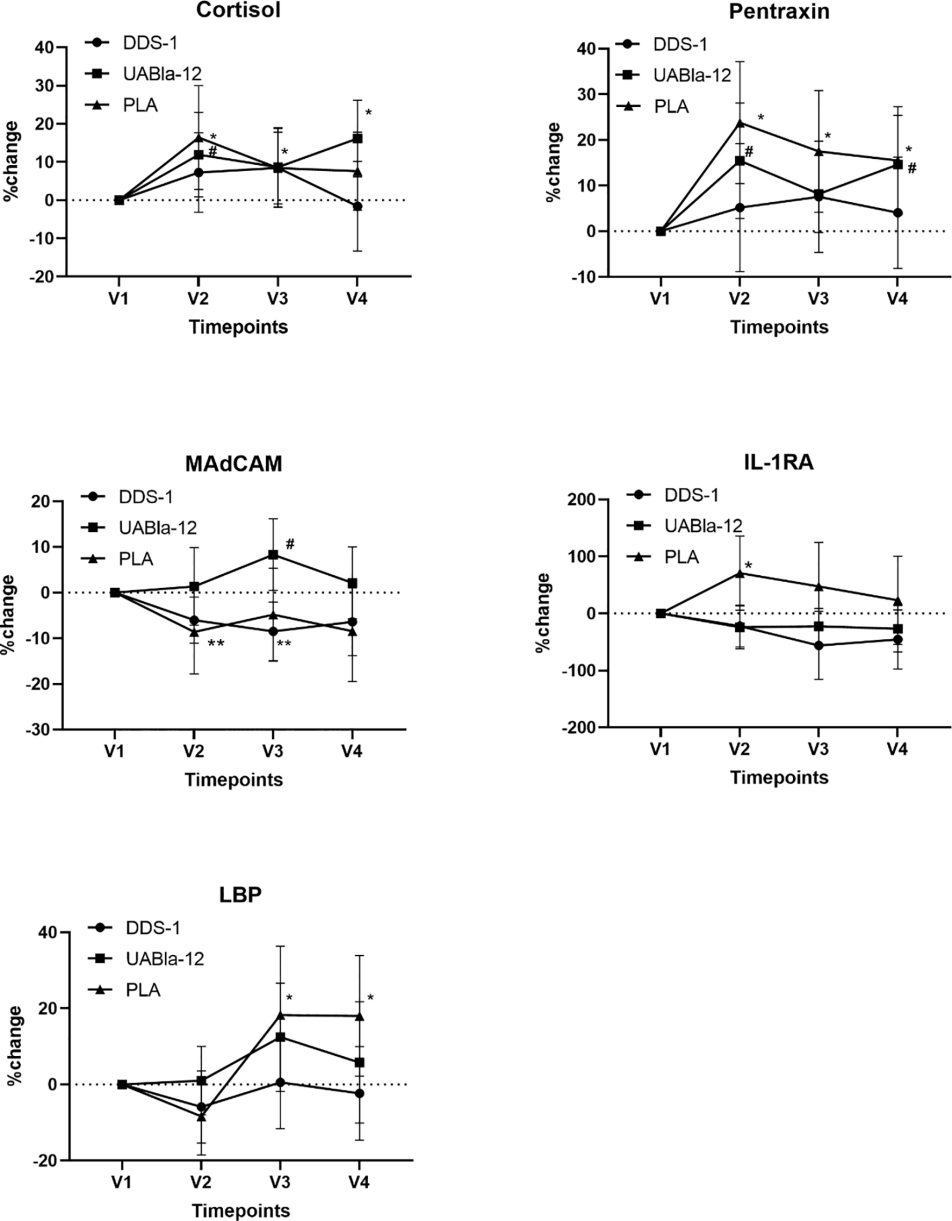
Changes in the concentration of serum analytes over the course of the study. Significantly larger within group changes in the placebo group are evident in all analytes from V1 to V2. Data are % change and 95% CI. The large changes in these analytes while adhering to typical night-day sleep-wake cycle were greater than the effect of nightshift on indices of stress, the acute phase response, serum cytokines and intestinal integrity markers. *significant change from V1 in the placebo group, ^#^significant change from V1 in UABla-12 group. **significance chance from V1 in the DDS-1 group.

The authors apologize for this error and state that this does not change the scientific conclusions of the article in any way. The original article has been updated.

In the published article, there was also an error in affiliation 3. Instead of “United Agricultural Services (UAS) Laboratories, Windsor, WI, United States”, it should be “UAS Laboratories, Windsor, WI, United States”.

The authors apologize for this error and state that this does not change the scientific conclusions of the article in any way. The original article has been updated.

