# Supplementary figures and images for: Probiotics, Anticipation Stress, and the Acute Immune Response to Night Shift
Source: Front Immunol. 2021 Jan 28;11:599547. doi: 10.3389/fimmu.2020.599547 (PMC7877220; doi:10.3389/fimmu.2020.599547)

**Supplementary Figure 1**

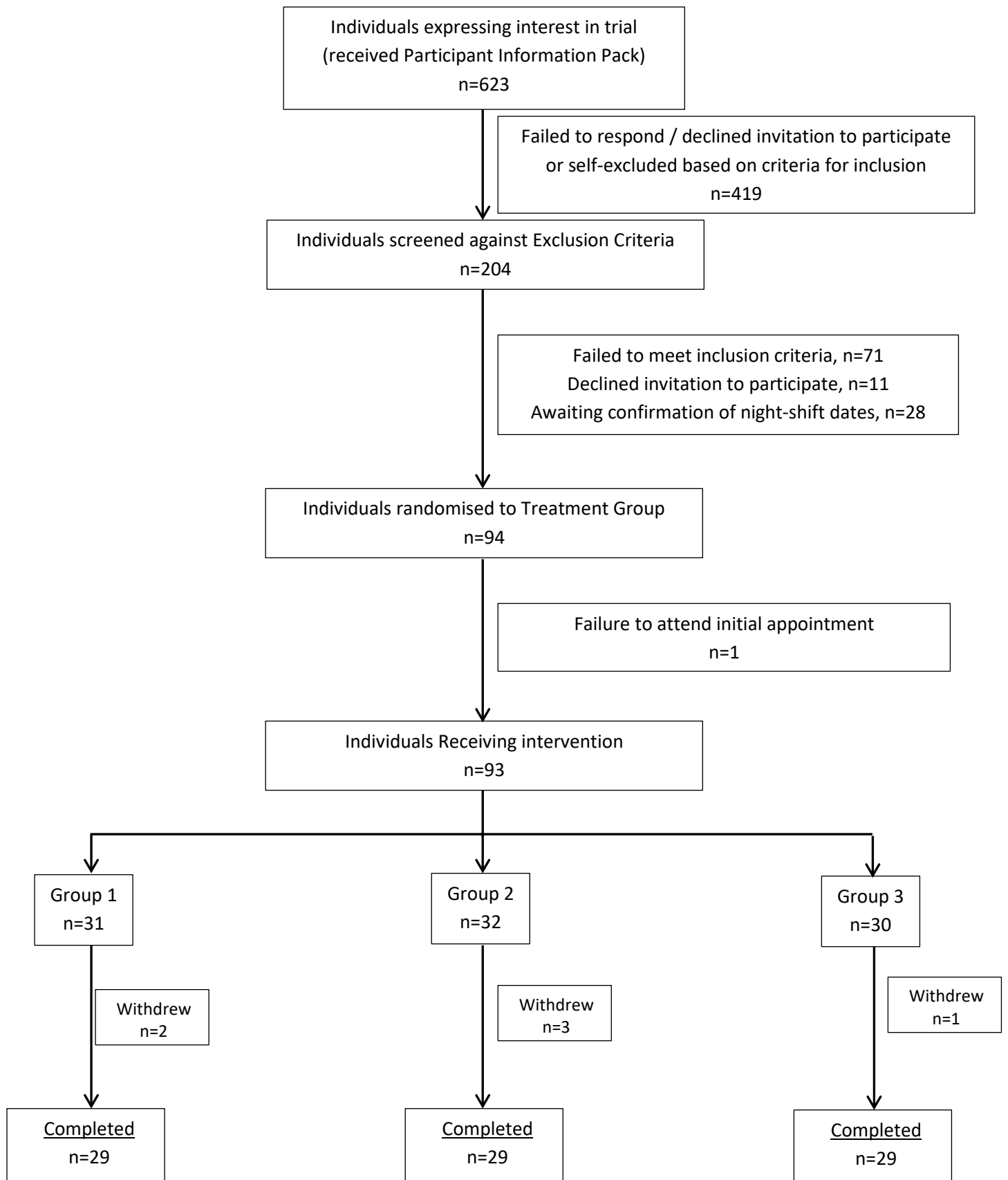

Supplement: Supplementary file 1 [file Image_1.pdf]
